# Tumor Necrosis Factor-Alpha Inhibitor-Induced Sarcoid-Like Reaction in a Patient With Juvenile Idiopathic Arthritis: A Case Report

**DOI:** 10.7759/cureus.84970

**Published:** 2025-05-28

**Authors:** Thanda Aung, Marisa Prasanpanich, Avery Kaplan, Kaitlin Eblen, Gregory A Fishbein

**Affiliations:** 1 Rheumatology, University of California Los Angeles David Geffen School of Medicine, Los Angeles, USA; 2 Pathology, King Chulalongkorn Memorial Hospital, Thai Red Cross Society, Bangkok, THA; 3 Pathology and Laboratory Medicine, University of California Los Angeles David Geffen School of Medicine, Los Angeles, USA

**Keywords:** anti-tnf-α, chronic juvenile rheumatoid arthritis, granuloma, hilar lymphadenopathy, sarcoid

## Abstract

Tumor necrosis factor-alpha (TNF-α) inhibitors are effective biologic disease-modifying antirheumatic drugs (bDMARDs) commonly used to treat various inflammatory conditions. While generally considered safe, these agents have been associated with paradoxical immune-mediated adverse effects, including sarcoid-like reactions. We present the case of a 24-year-old male with juvenile idiopathic arthritis (JIA) who developed pulmonary manifestations of a sarcoid-like reaction while on adalimumab therapy. The patient presented with progressive dyspnea, and imaging revealed diffuse bilateral ground-glass opacities with hilar lymphadenopathy. Endobronchial ultrasound-guided biopsy confirmed non-necrotizing granulomas with giant cells and eosinophils, consistent with a sarcoid-like reaction. Following discontinuation of adalimumab and initiation of methotrexate and prednisone therapy, the patient showed significant clinical and radiological improvement. This case highlights the importance of considering paradoxical drug reactions in patients on TNF-α inhibitors who present with respiratory symptoms and emphasizes the need for prompt diagnosis and appropriate management.

## Introduction

Sarcoidosis is a multi-system inflammatory disorder characterized by the formation of non-necrotizing granulomas in various organs. While its etiology remains unclear, both genetic predisposition and environmental factors are believed to contribute to its pathogenesis [1]. Clinical manifestations are diverse, ranging from general symptoms such as fatigue, fever, and weight loss to more specific presentations, including musculoskeletal complaints and respiratory symptoms. Respiratory involvement, which occurs in 30% to 53% of patients, may manifest as cough, dyspnea, and chest pain [1]. Diagnostic imaging typically reveals calcified lymph nodes, perilymphatic distribution of pulmonary micronodules, alveolar consolidations, or diffuse ground-glass opacities. Pulmonary function tests (PFTs) aid in diagnosis and severity assessment, while histological confirmation is obtained through endobronchial and transbronchial lung biopsies or endobronchial ultrasound (EBUS).

Biologic disease-modifying antirheumatic drugs (bDMARDs), particularly tumor necrosis factor-alpha (TNF-α) inhibitors, have revolutionized the treatment of various inflammatory rheumatological conditions, including rheumatoid arthritis and juvenile idiopathic arthritis [2]. Despite their generally favorable safety profile, with increased infection risk being the most common concern, TNF-α inhibitors have been associated with paradoxical immune-mediated adverse effects [3]. Among these, sarcoid-like reactions have been documented with etanercept, infliximab, and adalimumab [3,4].

Here, we present a case of adalimumab-induced sarcoid-like pulmonary reaction in a patient with juvenile idiopathic arthritis, highlighting the diagnostic challenges and therapeutic approach to this rare but significant adverse effect.

## Case presentation

A 24-year-old male with a long-standing history of juvenile idiopathic arthritis (JIA), initially diagnosed during childhood in Mexico, established care at our institution several years after his diagnosis. His medical history was notable for a single episode of anterior uveitis, which had been successfully treated with an oral prednisone taper. The patient's JIA was initially managed with a combination of methotrexate and etanercept, but his treatment regimen was subsequently transitioned to adalimumab. His adalimumab dosing schedule began with 40 mg subcutaneous injections every two weeks and was later adjusted to every three weeks as his disease came under control. The patient had maintained excellent disease control with no apparent treatment-related adverse effects throughout eight years of adalimumab therapy. He has no other medical conditions, and his only additional medication is over-the-counter artificial tears.

The patient presented to the Emergency Department reporting a two-month history of progressively worsening shortness of breath. Ten days prior to this presentation, he had sought medical attention at another healthcare facility for similar symptoms. At that time, his oxygen saturation was measured at 91% on room air, and chest radiography revealed bilateral pulmonary infiltrates. He was diagnosed with respiratory syncytial virus (RSV) pneumonia and discharged on a combination antibiotic regimen of amoxicillin-clavulanate and azithromycin.

Despite adherence to the prescribed antibiotic therapy, the patient experienced worsening dyspnea, developed clear sputum production, and experienced a single episode of hemoptysis. He denied fever or other constitutional symptoms throughout this period. His last adalimumab dose had been administered three weeks prior to presentation, having been temporarily discontinued following his pneumonia diagnosis. The patient reported no recent travel history and had no pets at home. He worked as a chef and had no history of exposure to dust, fumes, or chemicals in the workplace. He was a non-smoker.

Upon admission, empiric treatment with ceftriaxone and azithromycin was initiated for presumed pneumonia. Laboratory evaluation revealed a normal complete blood count, with the notable exception of peripheral eosinophilia (peak count 1.06 × 10^9/L) - a finding of particular interest in a patient with no prior history of asthma or atopic conditions. A comprehensive metabolic panel was unremarkable. Inflammatory markers were elevated, with an erythrocyte sedimentation rate of 36 mm/hr and C-reactive protein of 4.7 mg/L. Tuberculosis testing using QuantiFERON gold (QIAGEN, Venlo, The Netherlands) yielded negative results. An initial autoimmune serologic panel, including antinuclear antibodies (ANA), anti-cyclic citrullinated peptide (anti-CCP), anti-neutrophil cytoplasmic antibodies (ANCA), and rheumatoid factor (RF), returned negative. The patient's angiotensin-converting enzyme (ACE) level was markedly elevated at 110 U/L. 1,25-dihydroxyvitamin D levels were within normal limits (Table 1).

**Table 1:** Key laboratory abnormalities ACE: Angiotensin-converting enzyme; ESR: Erythrocyte sedimentation rate; CRP: C-reactive protein; ANA: Antinuclear antibody; RF: Rheumatoid factor; anti-CCP: Anti-cyclic citrullinated peptide; C-ANCA: Cytoplasmic anti-neutrophil cytoplasmic antibodies; P-ANCA: Perinuclear anti-neutrophil cytoplasmic antibodies; MTB: Mycobacterium tuberculosis; H: high; ELISA: Enzyme-linked immunoabsorbent assay.

Pertinent Lab Data	Patient's Lab Values	Reference Range
White Blood Cell Count (1/uL)	8.19	4.16 - 9.95 x10E3
Red Blood Cell Count (1/uL)	5.07	4.41 - 5.95 x10E6
Hemoglobin (g/dL)	14.7	13.5 - 17.1
Hematocrit (%)	43.9	38.5 - 52.0
Mean Corpuscular Volume (fL)	86.6	79.3 - 98.6
Mean Corpuscular Hemoglobin (MCH) (pg)	29	26.4 - 33.4
MCH Concentration (g/dL)	33.5	31.5 - 35.5
Red Cell Distribution Width - SD (fL)	38.4	36.9 - 48.3
Red Cell Distribution Width - CV (%)	12.1	11.1 - 15.5
Platelet Count, Auto (1/uL)	301	143 - 398 x10E3
Mean Platelet Volume (fL)	9.3	9.3 - 13.0
Absolute Nucleated RBC Count (1/uL)	0	0.00 - 0.00 x10E3
Neutrophil Abs (1/uL)	4.88	1.80 - 6.90 x10E3
Absolute Lymphocyte Count (1/uL)	1.23	1.30 - 3.40 x10E3
Absolute Mono Count (1/uL)	0.81	0.20 - 0.80 x10E3
Absolute Eos Count (1/uL)	1.06 (H)	0.00 - 0.50 x10E3
Absolute Baso Count (1/uL)	0.08	0.00 - 0.10 x10E3
Absolute Immature Gran Count (1/uL)	0.13	0.00 - 0.04 x10E3
ACE (U/L)	110 (H)	16 - 85
ESR (mm/hr)	36 (H)	<=12
C-Reactive Protein (mg/dL)	4.7 (H)	<0.8
Vitamin D, 1,25-DiOH (pg/mL)	37.5	19.9-79.3
ANA Ab Titer	<1:40	<1:40
Rheumatoid Factor (IU/mL)	<10	<14
Cyclic Citrulline Ab IgG (units)	5	0 - 19
C-ANCA (titer)	<1:20	<1:20
P-ANCA (titer)	<1:20	<1:20
MTB-QuantiFERON-Gold ELISA	Negative	Negative

**Figure 1:**
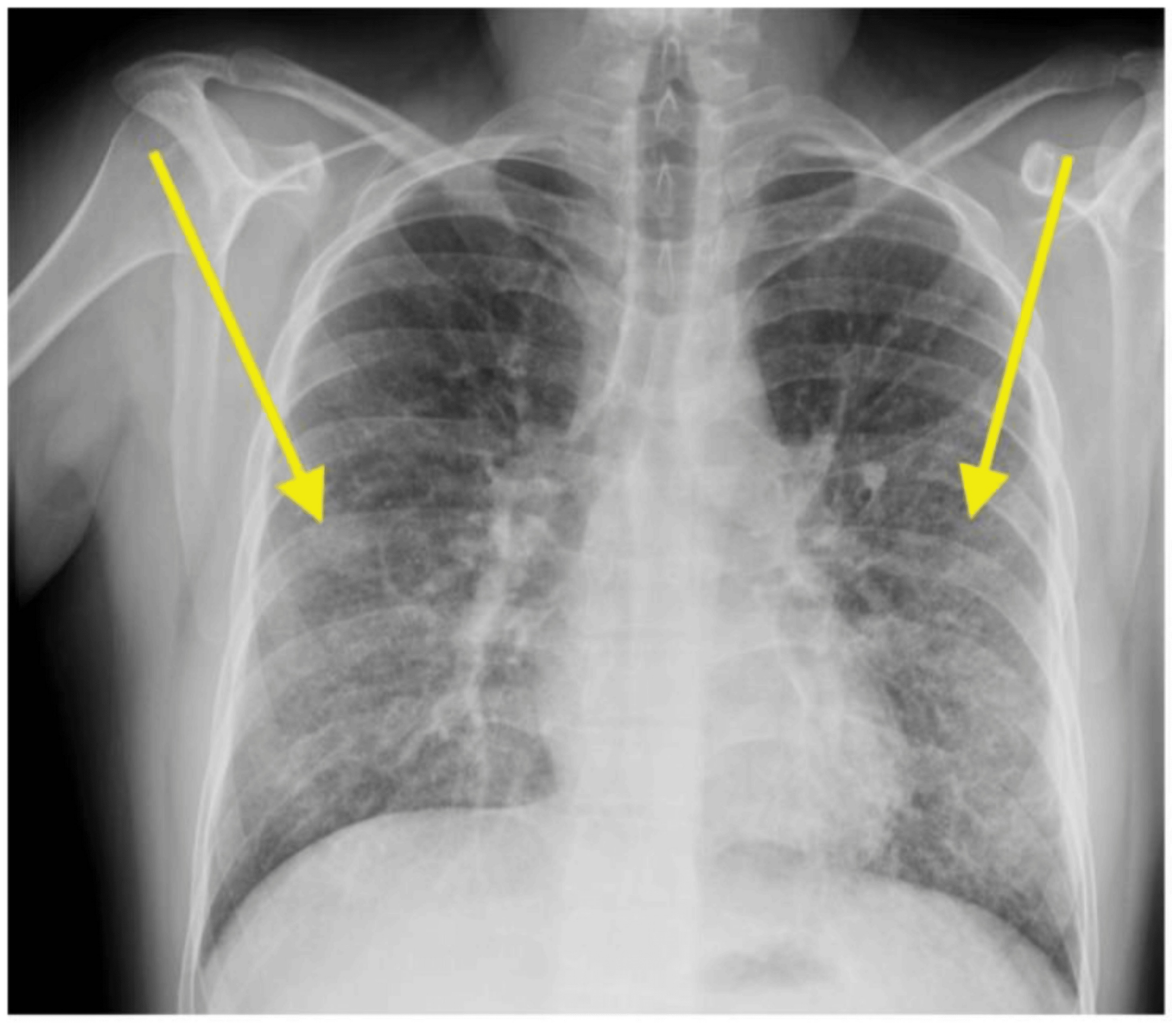
Chest radiography demonstrating diffuse bilateral mid to lower zone lung opacities (yellow arrows)

**Figure 2:**
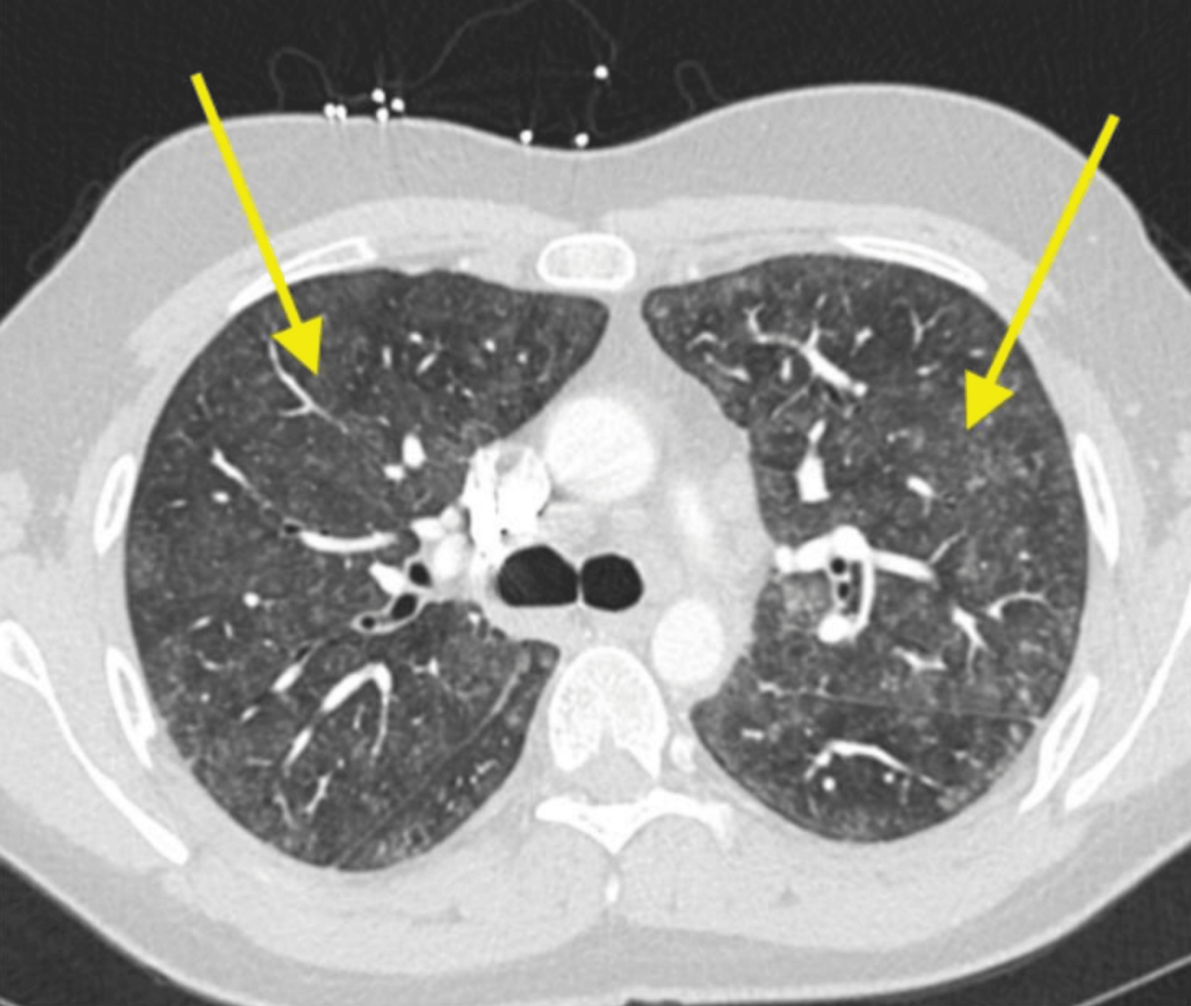
Chest CT showing diffuse centrilobular ground glass nodularity and bibasilar reticulations (yellow arrows)

Chest radiography demonstrated diffuse bilateral mid to lower zone lung opacities, initially interpreted as possible atypical pneumonia (Figure 1). Subsequent chest computed tomography (CT) revealed diffuse centrilobular ground-glass nodularity and bibasilar reticulations, without evidence of pulmonary embolism (Figure 2). The radiologic differential diagnoses included opportunistic infection, given the patient's immunosuppressed state, and immune-mediated disorders such as connective tissue disease-related interstitial lung disease, hypersensitivity pneumonitis, organizing pneumonia (e.g., cryptogenic organizing pneumonia), and drug reaction.

**Figure 3:**
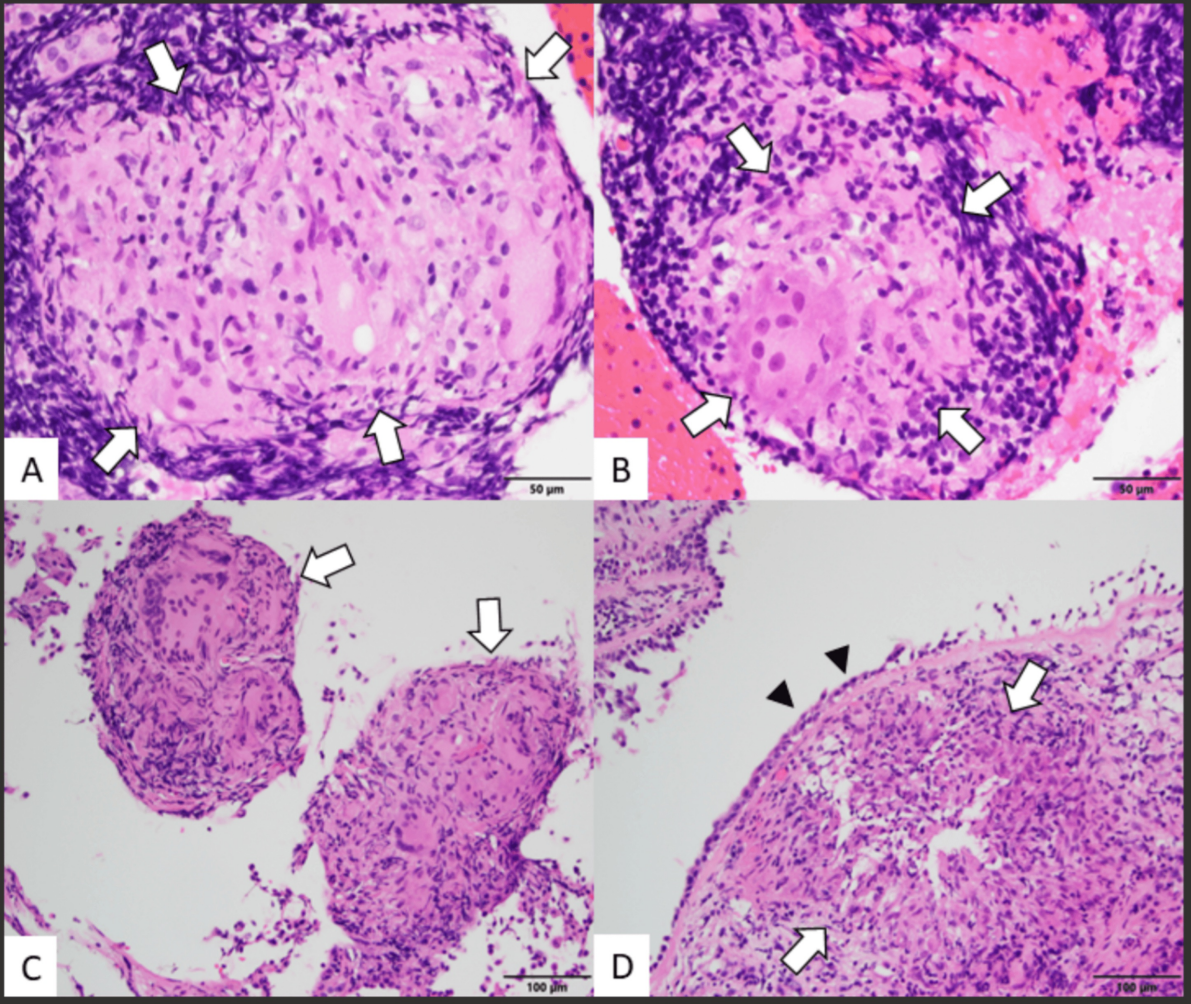
Histopathological features A-B: Well-formed, non-necrotizing granulomas (arrows) involving the mediastinal lymph nodes, Hematoxylin and Eosin, 400x magnification.  C: Biopsy from the left lower lobe of the lung revealed well-formed, non-necrotizing granulomas (arrows), Hematoxylin and Eosin, 200x magnification. D: Left mainstem bronchial biopsy demonstrated non-necrotizing granulomatous inflammation (arrows) beneath the respiratory epithelium (arrow heads), Hematoxylin and Eosin, 200x magnification.

The patient underwent endobronchial ultrasound (EBUS) with transbronchial biopsy, which revealed non-necrotizing granulomas with giant cells and eosinophils, findings consistent with a sarcoid-like reaction (Figure 3). Based on these diagnostic results, adalimumab therapy was discontinued, and treatment was initiated with a combination of methotrexate and prednisone. The prednisone regimen began at 40 mg daily and was gradually tapered to a maintenance dose of 5 mg daily over a three-month period. Concurrently, oral methotrexate was initiated at 7.5 mg weekly and carefully titrated upward to a target dose of 20 mg weekly.

**Figure 4:**
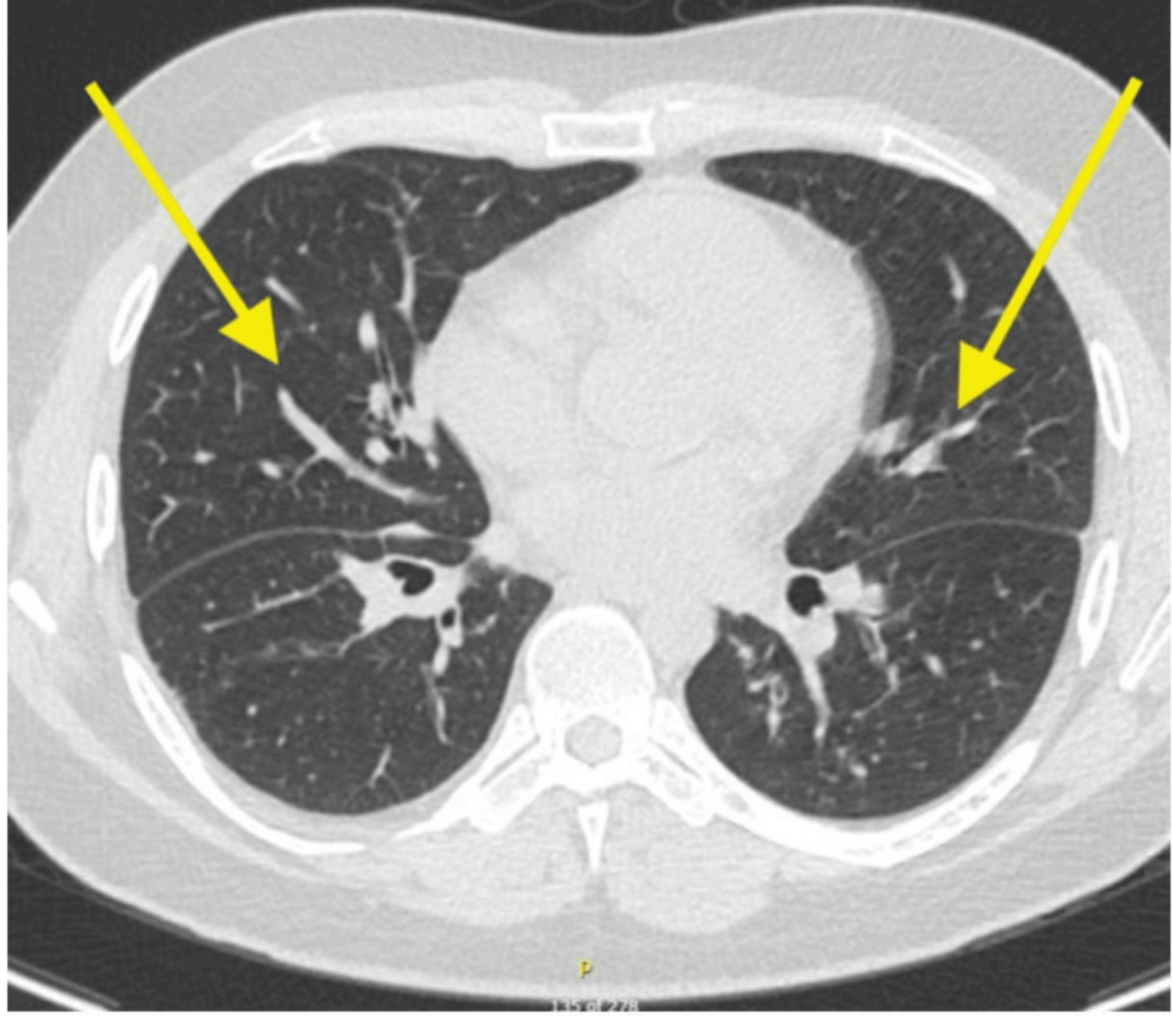
Chest high-resolution CT at the 6-month follow-up showing diffuse bronchial wall thickening (yellow arrows) and nearly resolved faint centrilobular ground-glass attenuation

The patient demonstrated an excellent response to this therapeutic approach, with progressive resolution of his dyspnea. Follow-up high-resolution CT imaging performed six months after treatment initiation showed significant improvement, with near-complete resolution of the previously observed centrilobular ground-glass attenuation (Figure 4). Eosinophilia was resolved within 6 weeks of discontinuation of adalimumab.

## Discussion

This case illustrates a TNF-α inhibitor-induced sarcoid-like reaction with pulmonary involvement in a patient with well-controlled juvenile idiopathic arthritis on adalimumab. The diagnosis is supported by the temporal relationship between treatment and symptoms, characteristic imaging findings, histopathological confirmation of non-necrotizing granulomas, and improvement after drug discontinuation.

According to Daïen et al. (2013), adalimumab accounts for 26% of TNF-α antagonist-induced sarcoidosis cases, with etanercept (42%) and infliximab (29%) comprising the remainder [5]. Pulmonary involvement, occurring in 66% to 74% of cases, represents the most common manifestation [6], with onset ranging from weeks to years after treatment initiation [7]. The peripheral eosinophilia and elevated ACE level observed in our patient are consistent with sarcoidosis, with the latter serving as a useful diagnostic marker [8]. Granulomatous lesions containing eosinophils further support this association.

TNF-α inhibitors paradoxically induce granulomatous reactions despite their anti-inflammatory properties. The exact mechanism is not fully understood. One proposed mechanism is the "compensatory immunological switch". By inhibiting TNF-α, there may be an upregulation of other pro-inflammatory pathways, such as interferon-gamma (IFN-γ) and interleukin-12 (IL-12), which can promote granuloma formation [9-11]. This shift in cytokine balance may lead to the development of non-caseating granulomas, characteristic of sarcoidosis [9-11]. Additionally, the paradoxical reaction may be a result of immune dysregulation. TNF-α plays a crucial role in maintaining immune homeostasis, and its inhibition can disrupt this balance, leading to aberrant immune responses and granuloma formation [11,12]. Beyond pulmonary involvement (74%), lymph node involvement (46%) and cutaneous lesions (25%) are frequently reported [6]. The variable timeframe from treatment initiation to sarcoidosis development necessitates ongoing vigilance.

Treatment centers on discontinuation of the offending agent, associated with complete or partial resolution in approximately 87% of cases [10]. Systemic corticosteroids (prednisone 0.5-1 mg/kg/day) serve as first-line therapy for significant organ involvement, with a gradual 3-6-month taper guided by British Thoracic Society guidelines [13]. Our patient responded well to an initial 40 mg dose, tapered to 5 mg daily maintenance over three months. For long-term or steroid-intolerant patients, steroid-sparing options include methotrexate (10-25 mg weekly), particularly useful for concurrent inflammatory arthritis; azathioprine (50-200 mg daily); and hydroxychloroquine (200-400 mg daily), effective for cutaneous and joint manifestations.

Regular monitoring should include clinical assessment, follow-up chest imaging, serial pulmonary function tests, and inflammatory markers. For patients requiring continued biologic therapy, switching to a different TNF-α inhibitor carries recurrence rates up to 60% [10]. Transitioning to biologics with alternative mechanisms (IL-6 inhibitors, IL-17 inhibitors, or Janus kinase (JAK) inhibitors) is generally preferred. Treatment typically spans 6-12 months, with possible maintenance therapy for recurrent or severe cases. Ongoing vigilance for fibrotic changes, pulmonary hypertension, or other sequelae is warranted. Ramos-Casals et al. (2010) analyzed 233 cases of autoimmune diseases induced by TNF-targeted therapies, emphasizing the importance of monitoring for these paradoxical immune reactions [14]. Our patient showed significant clinical and radiological improvement with combined methotrexate and prednisone, demonstrating the efficacy of this approach.

## Conclusions

TNF-α inhibitor-induced sarcoid-like reactions represent an important paradoxical adverse effect that clinicians should recognize when managing patients on biologic therapy. Our case of pulmonary manifestations in a juvenile idiopathic arthritis patient treated with adalimumab contributes to the growing literature on this phenomenon. Diagnosis depends on clinical suspicion, comprehensive evaluation including imaging and histopathology, and exclusion of infectious causes. Effective management involves discontinuing the implicated TNF-α inhibitor and implementing appropriate anti-inflammatory therapy based on disease severity. For significant pulmonary involvement, systemic corticosteroids with methotrexate as a steroid-sparing agent proved effective in our patient. The favorable outcome underscores the importance of prompt recognition and intervention, while suggesting the need for research into risk factors and optimized treatment strategies.
